# Antineutrophil Cytoplasmic Antibodies Associated With Infective Endocarditis

**DOI:** 10.1097/MD.0000000000002564

**Published:** 2016-01-22

**Authors:** Vincent Langlois, Anais Lesourd, Nicolas Girszyn, Jean-Francois Ménard, Hervé Levesque, Francois Caron, Isabelle Marie

**Affiliations:** From the Department of Internal Medicine, Institute for Biochemical Research, IFRMP, University of Rouen (VL, AL, NG, HL, IM); Department of Infectious diseases (FC); and Department of Biostatistics (J-F M), CHU Rouen, France.

## Abstract

To determine the prevalence of antineutrophil cytoplasmic antibodies (ANCA) in patients with infective endocarditis (IE) in internal medicine; and to compare clinical and biochemical features and outcome between patients exhibiting IE with and without ANCA.

Fifty consecutive patients with IE underwent ANCA testing. The medical records of these patients were reviewed.

Of the 50 patients with IE, 12 exhibited ANCA (24%). ANCA-positive patients with IE exhibited: longer duration between the onset of first symptoms and IE diagnosis (*P* = 0.02); and more frequently: weight loss (*P* = 0.017) and renal impairment (*P* = 0.08), lower levels of C-reactive protein (*P* = 0.0009) and serum albumin (*P* = 0.0032), involvement of both aortic and mitral valves (*P* = 0.009), and longer hospital stay (*P* = 0.016). Under multivariate analysis, significant factors for ANCA-associated IE were: longer hospital stay (*P* = 0.004), lower level of serum albumin (*P* = 0.02), and multiple valve involvement (*P* = 0.04). Mortality rate was 25% in ANCA patients; death was because of IE complications in all these patients.

Our study identifies a high prevalence of ANCA in unselected patients with IE in internal medicine (24%). Our findings further underscore that ANCA may be associated with a subacute form of IE leading to multiple valve involvement and more frequent renal impairment. Because death was due to IE complications in all patients, our data suggest that aggressive therapy may be required to improve such patients’ outcome.

## INTRODUCTION

Antineutrophil cytoplasmic antibodies (ANCA) directed against proteinase 3 (PR3) and myeloperoxydase (MPO) are strongly associated with primary systemic vasculitis, including granulomatosis with polyangiitis, microscopic polyangiitis, and eosinophilic granulomatosis with polyangiitis.^1,2^ Although, ANCA have also been described in other conditions, especially connective-tissue diseases, inflammatory bowel diseases, malignancies, and drug-induced vasculitis.^3^

Interestingly, ANCA have further been reported during the course of various infections, such as: viral (eg, hepatitis B and C, Epstein Barr virus, parvovirus B19, human immunodeficiency virus), bacterial (eg, *Staphylococcus*, *Streptococcus*, Bartonella, Gram-negative bacteria), fungal (eg, *Aspergillus*, Histoplasma), and parasitic (eg, Plamodium, *Entamoeba histolytica*) infections.^4^ Previous authors have speculated that infection-associated ANCA may be triggered by many immune dysfunction in response to microbial peptides leading to: upregulation of autoantigen genes, molecular mimicry between pathogen microorganisms and self-antigens, formation of neutrophil extracellular traps, interaction of pathogen microorganism components with toll-like receptors.^5–8^ More recently, the association between infective endocarditis (IE) and formation of ANCA has also been reported.^9–11^ To date, only a few series, however, have analyzed the prevalence and the outcome of ANCA-positive patients with IE, which prompted us to conduct the current retrospective study. Our aims were to: determine the prevalence of ANCA in patients with IE; and compare clinical and biochemical features and outcome between patients exhibiting IE with and without ANCA.

## PATIENTS AND METHODS

### Patients

From January 2010 to December 2014, 162 consecutive patients with IE were seen in the Department of Internal Medicine at the university of Rouen medical center. The definite diagnosis of IE was based on the modified Duke criteria.^12^ Ethical approval was obtained from the local ethical committee (Comité d’Ethique en recherche non interventionnelle for the Comité de protection des personnes de Haute-Normandie), and informed consent was obtained from all patients.

First, the medical records of patients with IE were reviewed for patients general characteristics at diagnosis: age and sex; comorbidities, such as arterial hypertension (cutoff 140/90 mm Hg), diabetes mellitus and cancer; previous medical history of: intravenous drug abuse, predisposing valvular disease (which was defined as having a native valve affected by regurgitation or stenosis), IE, endocavitary device, including pacemaker, cardioverter-defibrillator, left ventricular assist device, prosthetic material, that is, prosthetic heart valve, intravenous graft material, prosthetic joint, bone plate/screw, orthopedic rod; and immunosuppressive therapy for >30 days at time of IE diagnosis, including steroids, cytotoxic drugs, antitumor necrosis factor α, and rituximab.

All the patients had undergone the same routine clinical evaluation to investigate IE, as follows:

- Constitutional symptoms: fever ≥38 °C, chills, asthenia, weight loss
- Systemic manifestations, especially: congestive heart failure defined according to the New York Heart Association classification system,^13^ heart murmurs; vascular features, including arterial emboli, septic pulmonary infarctus, mycotic aneurysms, purpura, and Janeway lesions; rheumatologic signs: arthralgia, myalgia; and immunologic features, that are Osler nodes and renal impairment. In these patients, renal involvement was dichotomized into: acute renal failure and progressive renal failure. Kidney biopsies were designated as having the following patterns: mesangial proliferative glomerulonephritis (GN), focal necrotizing and crescentic GN, diffuse necrotizing and crescentic GN, focal proliferative or diffuse proliferative GN, membranoproliferative GN, thrombotic microangiopathy, and acute tubular injury; immunofluorescence pattern was determined, using fluorescein-tagged polyclonal rabbit antihuman antibodies to: C3, IgA, IgG, IgM, fibrinogen, and κ/λ-light chains. The outcome of renal involvement was determined as follows: complete resolution, characterized by normalization of serum creatinine values; persistent renal impairment characterized by persistent increase of serum creatinine 0.2 mg/L above baseline values; and end-stage renal involvement, requiring renal dialysis.^14^

Second, all patients had undergone transthoracic and/or transoesophageal echocardiography to detect: valvular impairment: aortic, mitral, tricuspid; localization and size of vegetations. Site of IE acquisition was defined following International collaboration on endocarditis^15^; and complications of IE such as paravalvular abscess and valvular perforation.

Third, the medical records of patients with IE were reviewed for laboratory characteristics at diagnosis of IE: C-reactive protein (mg/L), hemoglobin level (g/dL), leukocytosis (G/L), creatininemia (μmol/L), serum albumin (g/L); urinanalysis: hematuria, proteinuria; microbiological data: number of positive blood cultures, identification of pathogen microorganism.

Infective endocarditis features also included the timing of initial symptoms and patients’ hospitalization. Thus, IE was classified as: community-acquired IE, when IE was diagnosed at the time of admission or ≤48 hours of admission; health care-associated IE, when IE was diagnosed within 48 hours of admission in outpatients as follows: intravenous therapy, wound care or specialized nursing care at home ≤30 days before IE onset; attendance at hospital and/or intravenous chemotherapy ≤30 days before IE onset; hospitalization for ≥2 days in the 3 months days before IE onset; and residence in a long-term care facility; and nosocomial IE, when IE developed in patients hospitalized for >48 hours.^13,15^ Prosthetic valve IE was determined as an infection, involving: valve prothesis, reconstructed native heart valves whether a mechanical prosthesis and/or a bioprosthetic xenograft, stented or unstented, and/or a repaired native valve with implantation of an annular ring.^15,16^

Furthermore, all patients with IE received specific therapy. Data regarding patients’ therapy were reviewed for treatment used at any time during the course of IE, that is:

- Antimicrobial therapy
- Immunosuppressive therapy, including steroids and cytotoxic drugs
- And surgical procedures of IE complications

In addition, all patients underwent follow-up to determine the outcome of IE as follows: resolution; onset of complications; and in-hospital mortality (because of IE or to another cause than IE). Finally, the duration of hospital stay was also checked for all patients.

### Antineutrophil Cytoplasmic Antibodies

Antineutrophil cytoplasmic antibodies testing was performed using indirect immunofluorescence assay in ethanol-, formalin-, and methanol-fixed neutrophils. The ANCA staining pattern [cytoplasmic (c-ANCA) and perinuclear (p-ANCA)] was also determined by indirect immunofluorescence assays (Euroimmun, Lubeck, Germany). Furthermore, PR3 and MPO-ANCAs were detected by commercially available enzyme-linked immunosorbent assay (ELISA) (“anti-PR3-hn-hr IgG” and “anti-MPO-hn-hr IgG” Euroimmun, Lubeck, Germany).^17–20^ Values above 20 IU/mL were considered positive.

### Comparison of Antineutrophil Cytoplasmic Antibodies-positive and Antineutrophil Cytoplasmic Antibodies-negative Patients With Infective Endocarditis

We compared various features between the group of IE patients with and without ANCA, that is, median age, sex, comorbidities, previous medical history, constitutional, and systemic manifestations; biochemical findings; characteristics of IE; outcome of IE; and median duration of hospital stay.

### Statistical Analysis

Statistical analyses were conducted to assess the features of ANCA-associated IE. Patients were divided into 2 groups consisting of IE patients with and without ANCA.

For group comparison involving binary data, we used the Fisher exact test. Comparisons involving continuous data were performed using: Student test when distribution of variables was normal and Mann–Whitney test in other patients. The results were reported as the odds ratio (OR) and 95% confidence interval (95% CI); *P* values less than 0.05 were considered significant in all performed tests.

Finally, as regards variables with *P* values <0.1, we further proceeded with multiple logistic regression, using backward stepwise selection, to calculate multivariate OR (95% CI); the used level of significance was *P* < 0.05.

### Literature Review

In addition to the current study, we conducted a literature review of the Medline database (1966–2015) (National Library of Medicine, Bethesda, MD), reviewing articles of all languages, and we compared data from the current report with previously published data.

## RESULTS

Among the 162 consecutive patients with IE, findings of serum samples for ANCA were available for 50 patients. Indeed, from July 2013 to December 2014, all patients with IE (N = 50) systematically underwent ANCA testing.

Altogether, these 50 latter patients with IE were included in the study. Nevertheless, the characteristics of patients who did not undergo ANCA testing (from January 2010 to June 2013) were not different from those of patients who had ANCA testing (Table 1).

**TABLE 1:**
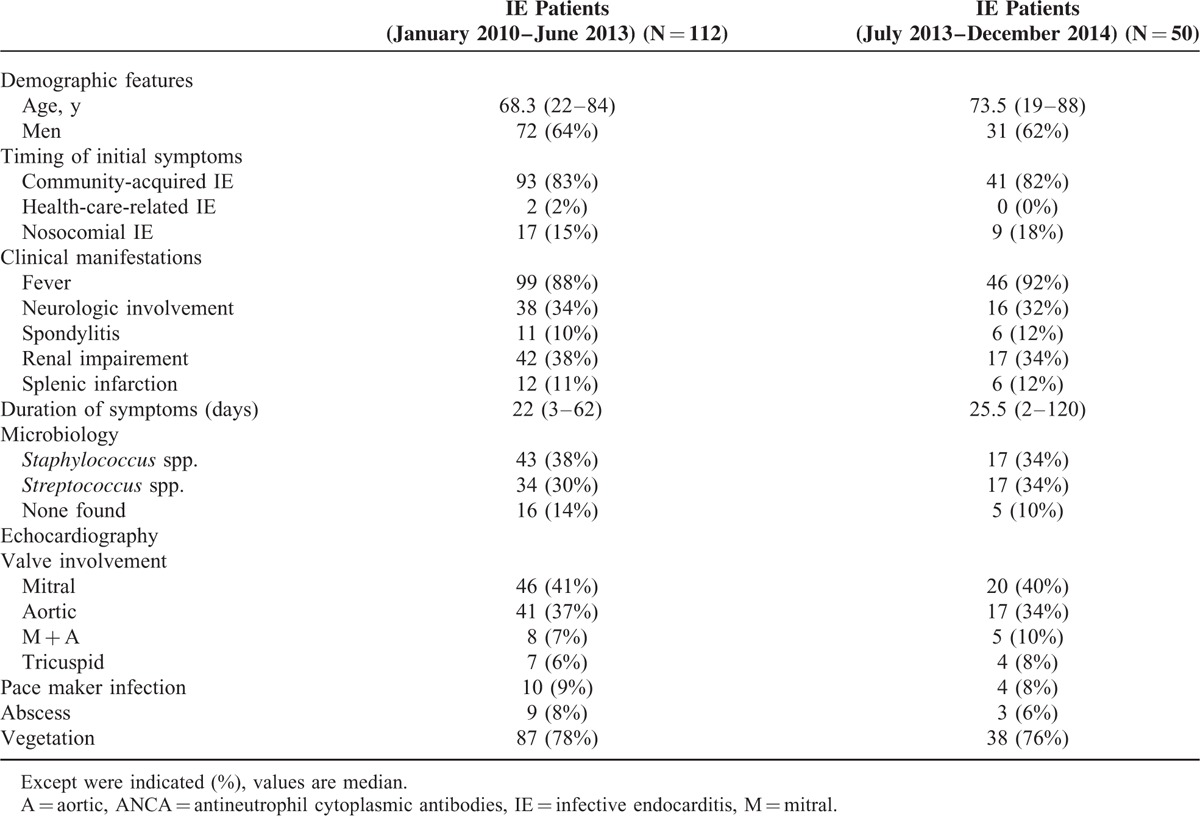
Characteristics of the 162 patients with infective endocarditis

All 50 patients had definite IE. Indeed, 2 of our patients, without valve vegetation on echocardiography, had valvular abscess. Furthermore, 2 other patients, without valve vegetation on echocardiography, exhibited cardiac marked uptake on ^18^F-fluorodeoxyglucose-positon emission tomography scanning. These 2 latter patients underwent cardiac valvular surgery; bacterial analysis of cardiac valvular specimens was positive for both cultures and *polymerase chain reaction*.

### Prevalence of Antineutrophil Cytoplasmic Antibodies in Patients With Infective Endocarditis

In our cohort of 50 patients with IE, ANCA were positive in 12 of them (24%).

Using ANCA staining pattern, patients exhibited c-ANCA (N = 6) and p-ANCA (N = 5).

Regarding ELISA test, patients had: PR3-ANCA (N = 4), MPO-ANCA (N = 1), PR3 + MPO-ANCA (N = 2). One of them exhibited high ELISA PR3 level without immunofluorescence positivity.

### Comparison of Characteristics Between Infective Endocarditis Patients With and Without Antineutrophil Cytoplasmic Antibodies

#### General Features

We failed to show any statistically significant differences between ANCA-positive and negative patients with IE regarding: age (*P* = 0.58), sex (*P* = 0.71); comorbidities: arterial hypertension (*P* = 0.11), diabetes mellitus (*P* = 0.74), and cancer (*P* = 1); previous history of: intravenous drug abuse (*P* = 1), predisposing condition on native valve (*P* = 0.70), IE (*P* = 1), pacemaker or cardioverter-defibrillator (*P* = 1), prosthetic heart valve (*P* = 0.47), and prosthetic joint (*P* = 1) (Table 2). Furthermore, we did not find any difference regarding previous immunosuppressive therapy (*P* = 0.24) between both groups of patients.

**TABLE 2:**
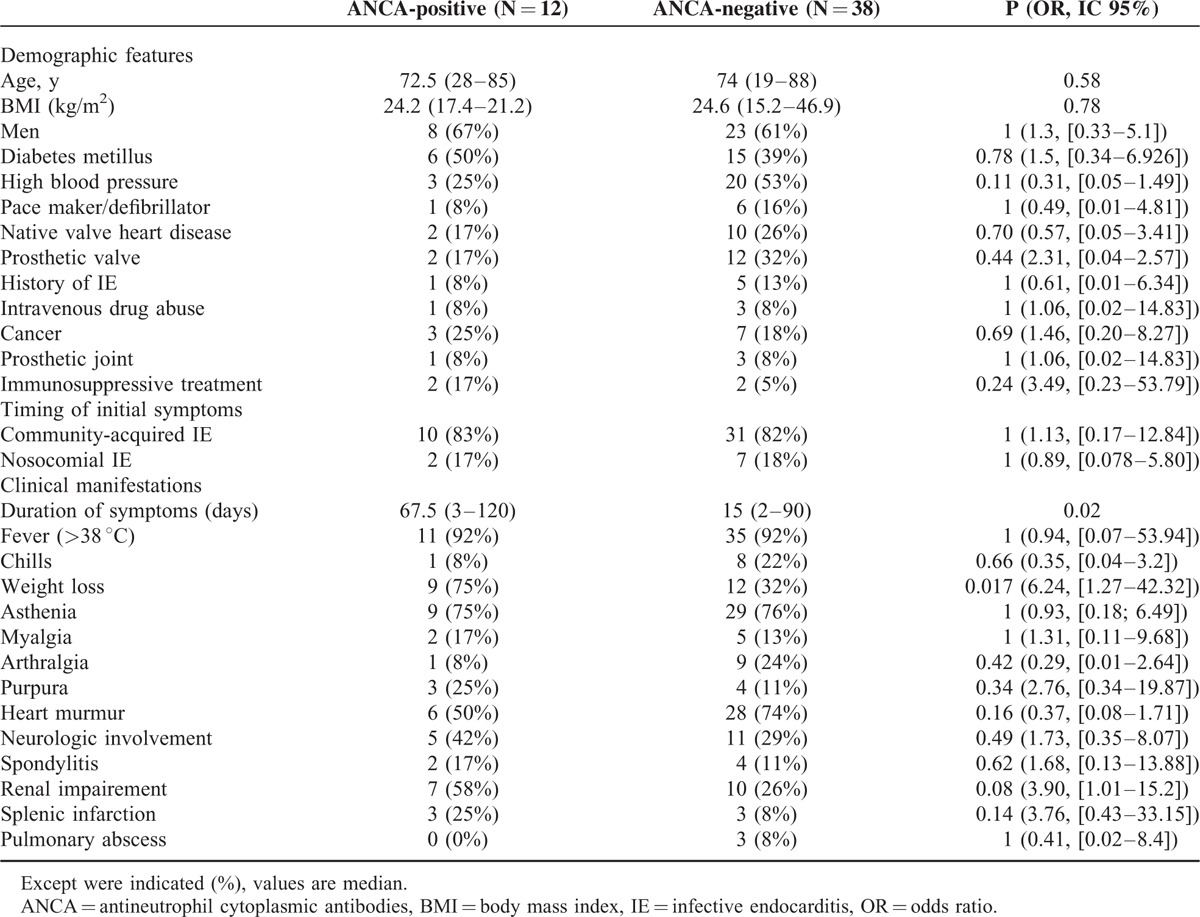
General and Clinical Characteristics in Infective Endocarditis Patients With and Without Antineutrophil Cytoplasmic Antibodies

#### Clinical Manifestations

The median duration between the onset of first symptoms and IE diagnosis was longer in ANCA-positive patients (67.5 versus 15 days; *P* = 0.02). Furthermore, ANCA-positive patients more often exhibited weight loss at IE diagnosis (*P* = 0.017).

Infective endocarditis patients with and without ANCA did not differ for: fever (*P* = 1), chills (*P* = 0.66), asthenia (*P* = 1); arterial emboli (*P* = 0.51), heart failure (*P* = 1), heart murmurs (*P* = 0.16); arthralgia (*P* = 0.42), myalgia (*P* = 0.65); purpura (*P* = 0.34), Janeway lesions (*P* = 1), Osler nodes (*P* = 0.43); spondylitis (*P* = 0.62); neurologic involvement (*P* = 0.49), including cerebral emboli (25% versus 18%), cerebral hemorrhage (4% versus 8%), cerebral mycotic aneurysms (8% versus 0%), and amyloid-like angiopathy (0% versus 3%); splenic infarction (0.14); and pulmonary abscess (*P* = 1) (Table 2).

Interestingly, renal impairment tended to be more frequent in patients with ANCA than in those without (58% versus 26%; *P* = 0.08), although not significantly so (Table 2). These 7 patients exhibited acute (N = 5) or rapidly progressive (N = 2) renal failure. Three of 7 patients who exhibited nephritic syndrome with hematuria (N = 1) underwent renal biopsy. In these 3 patients, histologic analysis of renal biopsy specimens showed: crescentic GN (N = 1). Glomerular inflammatory changes were diffuse in this patient, immunofluorescence studies showing presence of C3 and IgM. This patient progressed to end-stage renal disease, requiring renal dialysis; and diffuse proliferative GN with endocapillary proliferation without crescents or necrosis (N = 2), immunofluorescence studies being positive for C3 and IgG (N = 1) and IgM (N = 1). One of this patient exhibited complete renal resolution, whereas the other patient developed end-stage renal disease requiring renal dialysis. Regarding the 4 remaining patients who did not undergo renal biopsy, none exhibited nephrotic/nephritic syndrome; in these latter patients, the outcome of renal manifestations was as follows: complete renal resolution (N = 2) and persistent renal dysfunction (N = 2).

#### Laboratory Findings

As shown in Table 3, regarding biochemical tests, ANCA-positive patients with IE had lower median values of: C-reactive protein (*P* = 0.0009), hemoglobin (*P* = 0.02), and serum albumin (*P* = 0.0032).

**TABLE 3:**
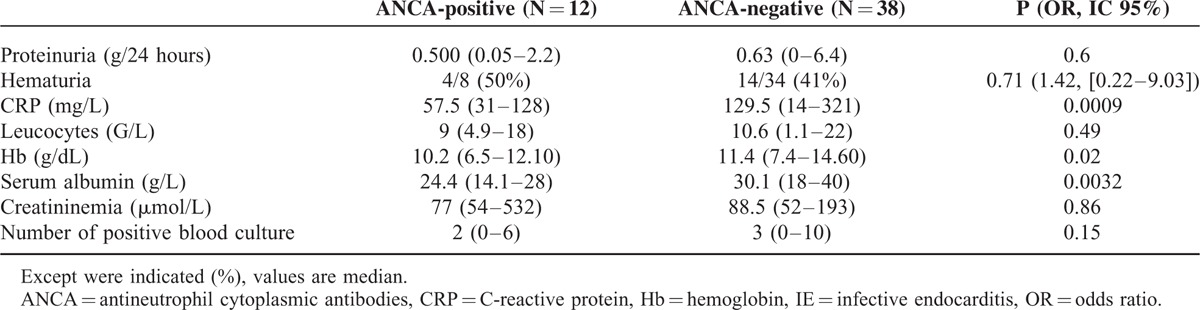
Biochemical Findings in Infective Endocarditis Patients With and Without Antineutrophil Cytoplasmic Antibodies

#### Characteristics of Infective Endocarditis

Patients with and without ANCA did not differ for prevalence of community-acquired IE, health care-associated IE or nosocomial IE (*P* = 1). The door-of-entry of IE was found in half of our ANCA-positive patients, as follows: colonic polyp (N = 2), intravenous drug abuse (N = 1), tooth extraction (N = 1), cardiac catheterization (N = 1), and chronic supra-pubic catheter (N = 1).

Antineutrophil cytoplasmic antibodies-positive patients with IE more frequently exhibited involvement of both aortic and mitral valves (*P* = 0.009). The prevalence of vegetations on echocardiography (*P* = 1) as well as the size of vegetations (*P* = 0.98) were not different between IE patients with and without ANCA (Table 4).

**TABLE 4:**
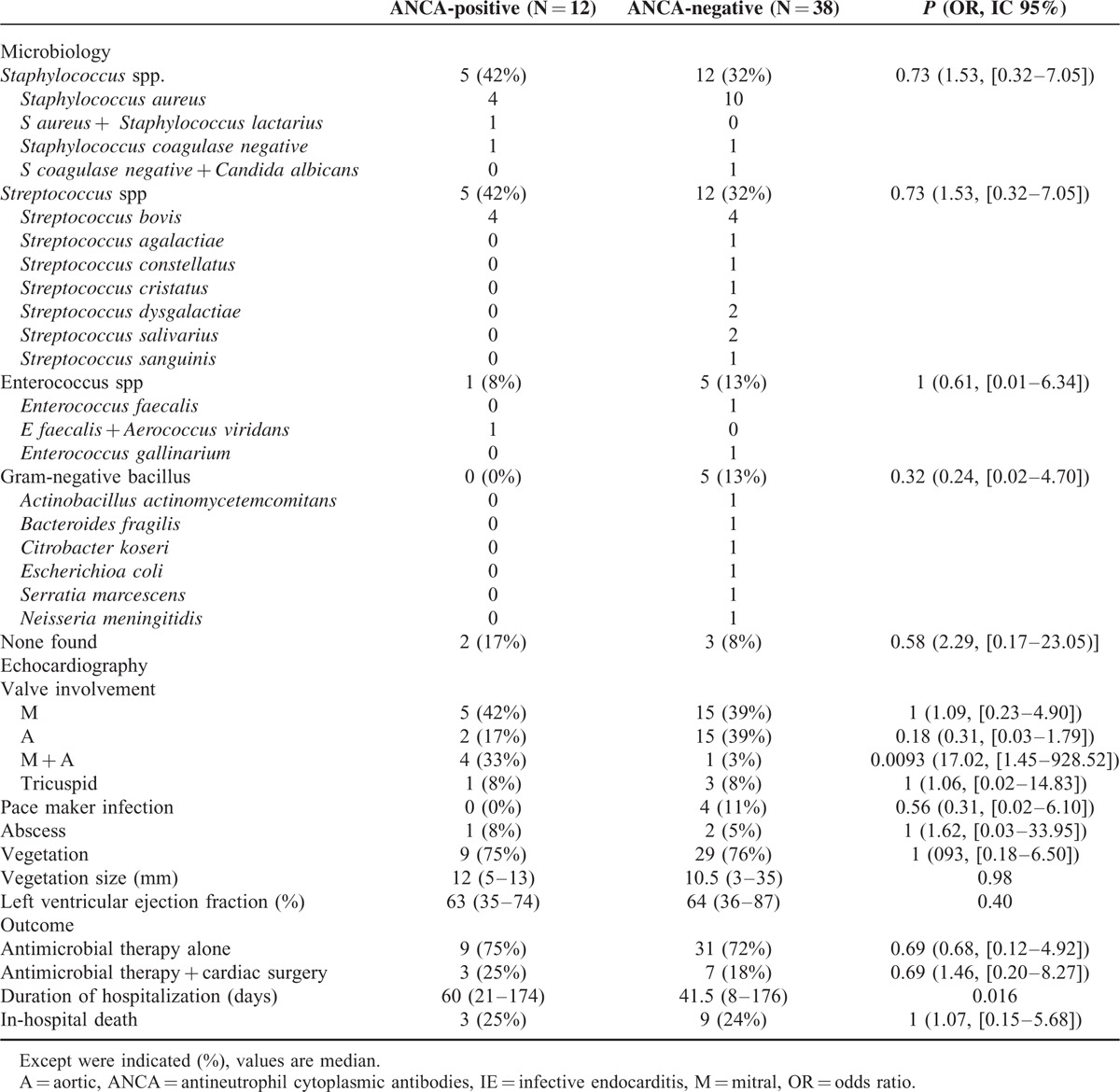
Microbiological and Echocardiographic Findings, Therapy, and Outcome of Infective Endocarditis Patients With and Without Antineutrophil Cytoplasmic Antibodies

On echocardiography, there was also no significant difference between patients with and without ANCA regarding the following IE complications: paravalvular abscess (*P* = 1); valvular perforation (*P* = 1) and cardiac dysfunction, as shown by similar median value of left ventricular ejection fraction (63% versus 64%; *P* = 0.40).

In addition, we failed to find differences between patients with and without ANCA regarding the causative pathogen microorganism of IE, that is, Staphylococcus spp. (*P* = 0.73), Streptococcus spp. (*P* = 0.73), Enterococcus spp. (*P* = 1), Gram-negative bacillus (*P* = 0.32), or absence of micro-organism (*P* = 0.58) (Table 4). Finally, the median number of positive blood cultures tended to be lower in the group of ANCA-positive patients with IE (2 versus 3; *P* = 0.15), although not significantly so.

#### Outcome of Infective Endocarditis

Therapy of IE did not differ between patients with and without ANCA for: antimicrobial therapy alone (75% versus 82%; *P* = 0.69) and combined therapy of antimicrobial agents and surgery procedures (25% versus 18%; *P* = 0.69). Surgical therapy consisted of: debridement associated with valvuloplasty (33% versus 43%) and heart valve replacement (67% versus 57%). Furthermore, 2 patients in ANCA-positive group also received immunosuppressive therapy [bolus of intravenous corticosteroids: 750 mg (N = 1) and 1 g N = 1)] because of initial diagnosis of systemic vasculitis. By contrast, no misdiagnosis was made in ANCA-negative group (*P* = 0.054).

Furthermore, in-hospital mortality was similar between IE patients with and without ANCA (25% versus 24%; *P* = 1). Death was directly because of IE complications: in the 3 patients with ANCA (25%): multiple organ failure (N = 2), end-stage renal failure (N = 1); and in the 9 patients without ANCA (24%): cerebral hemorrhage (N = 3), heart failure (N = 2), respiratory distress (N = 1), cardiopulmonary failure (N = 1), multiple organ failure (N = 2), and sudden death (N = 1). Finally, the median duration of hospital stay was higher in ANCA-positive patients (60 versus 41.5 days; *P* = 0.016).

### Results of Multiple Logistic Regression Analysis to Identify Infective Endocarditis Parameters Associated With Antineutrophil Cytoplasmic Antibodies

Longer duration of symptoms before diagnosis (OR: 1.47 [95% CI: 1.14–1.90]; *P* = 0.004) and multiple valve (mitral and aortic) involvement (OR: 1.31 [95% CI: 1.02–1.71]; *P* = 0.04) were significant factors for IE associated with ANCA.

## DISCUSSION

Infective endocarditis still remains a serious condition. In previous series, the clinical features, etiology, complications, and outcome have been documented in patients with IE, which resulted in most appropriate therapies, although mortality remains high in these patients.^16,21–30^ Interestingly, few investigators have described ANCA-associated IE. These authors have thus reported ANCA in 18% to 33% of patients with IE.^31,32^

To the best of our knowledge, the current study is one of the largest to evaluate the prevalence of ANCA in patients with IE. Among 50 unselected patients with IE, up to 24% had ANCA. Because IE is not an uncommon disease, our findings thus underscore that IE is an important cause of ANCAs in internal medicine. Although, the current study has, in fact, the following limitations: our study was done at tertiary hospital. Thus, our cohort may not reflect the features of overall patients with IE in the general population. Accordingly, our conclusions are applicable to IE patients admitted in Departments of Internal Medicine of tertiary hospitals. Although, this bias cannot be avoided, it has to be known;^27^ the possibility that ANCA might have been positive before IE onset cannot be ruled out, although our ANCA-positive patients did not have previous history of connective-tissue disease and/or systemic vasculitis; and follow-up sera were not tested systematically in all 12 IE patients with ANCA. In 3 of these patients who underwent ANCA testing at 3-month follow-up, ANCA testing, however, proved negative after successful antibiotic therapy of IE. Our latter findings reinforce the relationship between IE and ANCA in these patients. Altogether, our findings suggest that the identification of ANCA may be useful in IE to improve such patient's management.

In our literature search using the Medline database, we identified 50 well documented cases of ANCA-associated IE; most of them were case reports.^9–11,21,24,31–61^ Interestingly, we observed that IE patients with ANCA more often exhibited: c-ANCA than p-ANCA (84% versus 14%); and PR3 ANCA compared with MPO-ANCA and PR3 + MPO-ANCA (63% versus 17% versus 10%) (Table 4). The current study also highlights that patients with IE more often developed c-ANCA.

Mechanisms of ANCA-induction are still unclear in patients with IE. Previous authors have postulated that ANCA formation in IE may result from: autoantigen complementarity has been suggested one of the mechanisms breaking tolerance to ANCA antigens.^47,62^ The complementary peptide immunogen could be a microbial exogenous peptide.^47,62^ Several microorganisms (*Staphylococcus aureus*, *E histolytica*) have peptides homologous to cPR3; and may trigger an autoantibody response.^47,63^ It has been speculated that complementary peptides to PR3 could be beneficial to pathogens expressing cPR3 by binding to/neutralizing the antimicrobial properties of PR3 and MPO^64^; molecular mimicry between epitopes of self-antigens and antibodies to microorganisms.^47^ Lysosome-associated membrane protein-2 (LAMP-2) is a glycosylated type 2 membrane protein expressed on the membrane of neutrophil intracellular vesicles, containing PR3 and MPO.^47,65^ Anti-LAMP-2 ANCA cross react with the adhesive bacterial fimbrian protein Fim-H, which is detected in many Gram-negative bacteria; thus, previous investigators have suggested that anti-LAMP-2 ANCA may be because of molecular mimicry following Gram-negative bacteria (with Fim-H)^7,47^; neutrophil extracellular traps formation may result in the synthesis, in neutrophils, of antimicrobial proteins such as PR3 and MPO.^47^ It seems to play an important role in bacterial infection (Staphylococcus, Streptococcus, Enterococcus) control;^35,47,60^ and Toll-like receptors. The bacterial DNA hypomethylated motifs (ligands for Toll-like receptor 9) have been found to lead to the in vitro production of ANCA by B-lymphocytes.^11,47^

From a practical point of view, the identification of features of ANCA-associated IE appears to be crucial, to improve both early diagnosis and management in these patients. Nevertheless, to our knowledge, no predictive factors for ANCA-associated IE have been clearly defined to date.

In our literature review, we observed that ANCA-associated IE tended to occur more commonly in men (80% of patients); in these patients, the median age at IE diagnosis was 53.5 years^9–11,21,24,31–61^ (Table 5). The current study confirms these data, as many of our patients (67%) were men, although our data do not permit us to determine whether there is an association between sex and ANCA-associated IE. Furthermore, the median age of our patients with ANCA-associated IE (72.5 years) reflects, in part, the demographic features in the north-western area in France.

**TABLE 5:**
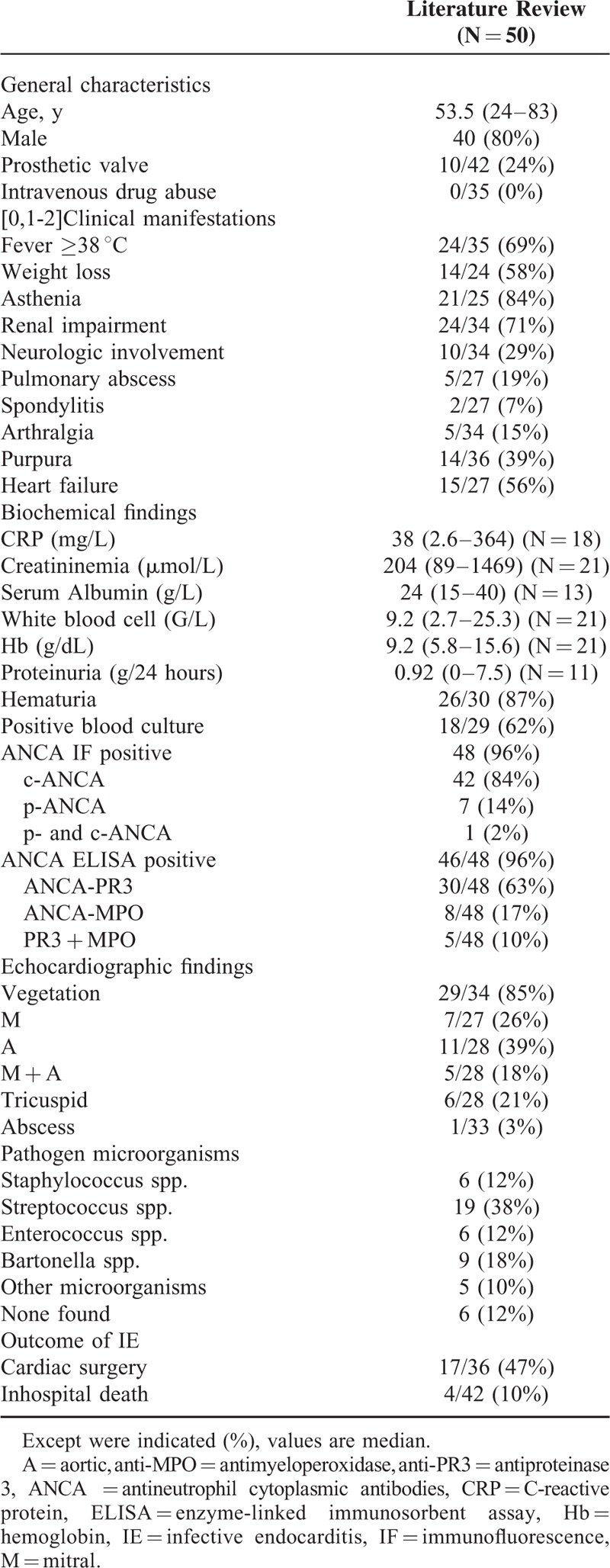
Literature Review^9–11,21,24,31–61^

In our review, we further found that the main clinical manifestations of ANCA-associated IE were: asthenia (84%), fever (69%), weight loss (58%), and renal impairment (71%) (Table 5).^9–11,21,24,31–61^ In this instance, IE patients with ANCA, compared with those without, more frequently presented: deterioration of general status with weight loss; and renal impairment (58% versus 26%). In 29 IE-associated biopsy proven GN, 8 patients (28%) had ANCA. Unfortunately, these investigators did not compare the clinical and histologic features of GN between IE patients with and without ANCA.^7^ In our 3 ANCA-positive patients with biopsy proven GN, patients exhibited diffuse proliferative GN (N = 2) and necrotizing/crescentic GN (N = 1). Our findings suggest that IE patients with ANCA should undergo systematic investigations to detect underlying life-threatening renal complications, although no definite conclusion can be drawn from our data. Our literature review, in fact, identified 24 case reports of documented ANCA-positive patients with IE exhibiting renal impairment.^11,24,37,39,40,42–45,47,48,50,51,54,57–59,61^ Twenty-one of these latter patients underwent renal biopsy showing: extracapillary GN with immune deposits (N = 5), pauci-immune GN (N = 4), segmental and focal necrotizing GN (N = 4), endocapillary GN with immune deposits (N = 3), interstitial nephritis (N = 1), chronic sclerotic GN (N = 1), both pauci-immune GN and interstitial nephritis (N = 2), and both endocapillary GN and interstitial nephritis (N = 1).^11,24,37,39,40,42–45,47,48,50,51,54,57–59,61^ Altogether, our data suggest that whether patients with ANCA-associated IE, develop commonly renal impairment, renal damage are nonspecific in these patients. Some damage could mimic ANCA-associated vasculitis renal attempts (pauci immune GN). On the contrary, in ANCA-negative IE, other authors reported that the most common biopsy finding was crescentic GN followed by endocapillary proliferative GN.^14,66,67^ Interestingly, in our experience, most ANCA-positive patients with IE had favorable outcome of renal impairment (N = 4/7). Thus, we suggest that renal biopsy may be performed in the population of ANCA-positive patients with IE, who do not exhibit improvement of renal function tests after initiation of appropriate antibiotic therapy, to detect other underlying condition responsible for renal dysfunction.

Another interesting finding in our study was that diagnosis at an early stage of IE was less frequent in the group of ANCA-positive patients, as shown by longer median interval between onset of first symptoms and IE diagnosis. We suggest that the presence of constitutional symptoms (weight loss, asthenia), renal impairment, and ANCA has resulted in the diagnosis at later stage of IE. In our patients, weight loss, asthenia, and low fever (≤38.5 °C) were indeed the only symptoms of IE in up to 17% of patients. It is thus crucial to maintain a low threshold for investigation of IE in these ANCA-positive patients to avoid underdiagnosis. Also, the onset of a new heart murmur is an important clinical sign for the diagnosis of IE at earlier stage in these patients.

Furthermore, the main differential diagnoses of these insidious forms of ANCA-positive IE are ANCA-associated vasculitis.^68,69^ Comparing previous and current reports of ANCA-associated IE and granulomatosis with polyangiitis,^69^ we have interestingly observed the following distinct features between both groups of patients, that is: pulmonary (13% versus 85%; *P* < 0.0001) and articular signs (13% versus 67%; *P* < 0.0001) were less common in ANCA-associated IE; fever (≥38 °C) (74% versus 50%; *P* < 0.0042) and weight loss were more frequent (64% versus 35%; *P* = 0.0023) in ANCA-associated IE; and up to 25% of our ANCA-associated IE patients presented splenic infarction, whereas splenic infarction is an uncommon complication of ANCA-associated vasculitis.^70–73^ On the contrary, we failed to find difference regarding renal involvement, cutaneous symptoms, or neurologic involvement between ANCA-positive patients with IE and patients with granulomatosis and polyangiitis (Table 6).

**TABLE 6:**
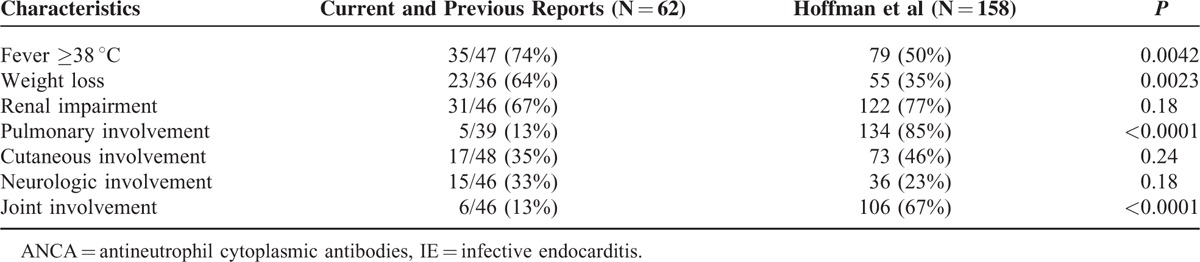
Characteristics and Organ Involvements in a Population With Antineutrophil Cytoplasmic Antibodies-associated Infective Endocarditis (Current Report, Literature Data^9–11,21,24,31–61^) Versus Granulomatosis With Polyangiitis Cohort^25,66^

Regarding biochemical tests, IE patients with ANCA more often developed inflammatory process than those without, as shown by lower serum albumin and inflammatory anemia. Overall, these findings suggest that ANCA-positive patients with IE more often exhibit a subacute form of IE, with prolonged inflammation, resulting in diagnosis of IE at a later stage in this group of patients.

As with IE in the general population, the main causative pathogen microorganisms in ANCA-positive patients with IE were Staphylococci and Streptococci.^11^ Our study, however, shows a higher prevalence of IE related to Staphylococcus than has previously been reported (42% versus 12%) in ANCA-positive patients.^37,42,45,49^ This discrepancy may be explained by the predominance of *S aureus*, which has increasingly been described in America and Europe.^74,75^

A potential door-of-entry of IE was found in only 50% of our ANCA-positive patients; these data may be explained by the fact that most of ANCA-positive patients had community-acquired IE (83%); of note, no patient had health-care-associated IE and 17% of patients had nosocomial IE. In this instance, the prevalence of community-acquired IE and nosocomial IE was similar in both groups of patients.

Culture-negative IE accounted for as high as 17% of our ANCA-positive patients. Our findings confirm previous authors’ data, reporting culture-negative IE in 12% of such patients.^31,43,48,58^ In this instance, we did not experience cases of ANCA-positive patients with IE related to Bartonella spp. On the contrary, in our literature review, we have noted an overrepresentation of IE related to Bartonella (mainly *Bartonella henselae* and *Bartonella quintana*) in ANCA-positive patients^31,44,50–55,59^. Indeed, Aslangul et al^34^ reported ANCA positivity in up to 60% of patients with Bartonella IE. These authors have postulated that Bartonella effects in endothelium with recruitment of macrophages and leukocytes associated with delayed leukocytes apoptosis may lead to ANCA production.^76^ These data suggest that ANCA-positive patients with IE, exhibiting negative blood cultures, should undergo Bartonella serology and/or research by polymerase chain reaction in heart valves (in those requiring cardiac surgery).

On echocardiography, presence of vegetations was detected in 75% of our ANCA-positive and -negative patients with IE. This prevalence was lower than the rate of 85% reported in this subgroup of patients, in our literature review^9–11,21,24,31–61^ (Table 5); vegetation sizes were also similar between ANCA-positive and ANCA-negative IE patients. Our findings suggest that vegetations features may not be predictive factors of ANCA-associated IE.

Interestingly, Chirinos et al^38^ have reported that valvular heart involvement in ANCA-associated vasculitis systematically affect the aortic valve. Thus, another main finding in our study was that IE patients with ANCA, compared with those without, more commonly exhibited both aortic and mitral valvular involvement. In our literature review, ANCA-positive patients with IE exhibited: isolated mitral (39%) or aortic (39%) valvular vegetations, and both mitral and aortic vegetation (18%).^9,51,52,57,59^ Thus, in our ANCA-positive patients, we suggest that diagnosis of IE at later stage and immunosuppression (related to older age and marked weight loss) may have resulted into increased bacterial mitral and aortic colonization. Our data are relevant as they suggest that the presence of multiple valve involvement may be a predictive marker of IE rather than systemic vasculitis in ANCA-positive patients. Because of the small number of ANCA-positive patients with IE, further investigations, however, are warranted to confirm our findings.

In addition, another finding in our study was that hospital stay was longer in our ANCA-positive patients with IE. Our data may be, in part, explained by the fact that these patients had diagnosis of IE at later stage, resulting in more severe forms of IE, including multiple valve involvement, renal impairment, and deterioration of general status with marked weight loss. Furthermore, the overall in-hospital mortality rate was 25% in our ANCA-positive patients with IE, which is higher than the rate of 10% reported in the literature;^37,43,52^ it is possible that this discrepancy may be related to: older age and higher prevalence of Staphylococcus infection in our cohort; and low rate of surgery (25%) in our patient, as early surgery is believed to be an effective treatment to decrease mortality in patients with IE.^77–79^ In the current study, only in-hospital follow-up, however, was available for our patients. Thus, we cannot exclude that IE patients with ANCA have a decreased late survival; in fact, because of multiple valve involvement, probability of valvular surgery may be higher at long-term follow-up in these patients, leading to increased morbidity and mortality of valvular surgery.^22^

In conclusion, our study identifies a high prevalence of ANCA in unselected patients with IE in internal medicine (24%). Our findings further underscore that ANCA may be associated with a subacute form of IE leading to multiple valve involvement and more frequent renal impairment. Because death was because of IE complications in all patients, our data suggest that aggressive therapy, including valvular surgery, may be required to improve such patients’ outcome; although, only randomized trials would specify whether valvular surgery may improve the prognosis of such patients.
